# Translating PUFA omega 6:3 ratios from wild to captive hibernators (*Urocitellus parryii*) enhances sex-dependent mass-gain without increasing physiological stress indicators

**DOI:** 10.1007/s00360-022-01437-6

**Published:** 2022-05-03

**Authors:** Monica Mikes, Sarah A. Rice, Doug Bibus, Alexander Kitaysky, Kelly L. Drew

**Affiliations:** 1 Department of Chemistry and Biochemistry, University of Alaska Fairbanks, Fairbanks, AK, USA; 2 Institute of Arctic Biology, University of Alaska Fairbanks, Fairbanks, AK, USA; 3 Lipid Technologies, LLC, Austin, MN, USA; 4 Department of Biology and Wildlife, University of Alaska Fairbanks, Fairbanks, AK, USA

**Keywords:** Hibernation, PUFA, Omega 3, Ground squirrel

## Abstract

Omega 3 polyunsaturated fatty acids (PUFAs) are well-documented for their influence on health and weight loss. Recent studies indicate omega 3 PUFAs may exert a negative impact on cellular stress and physiology in some hibernators. We asked if physiological stress indicators, lipid peroxidation and mass gain in Arctic Ground Squirrels (AGS) were negatively influenced by naturally occurring dietary omega 3 PUFA levels compared to omega 3 PUFA levels found in common laboratory diets. We found plasma fatty acid profiles of free-ranging AGS to be high in omega 3 PUFAs with balanced omega 6:3 ratios, while standard laboratory diets and plasma of captive AGS are high in omega 6 and low in omega 3 PUFAs with higher omega 6:3 ratios. Subsequently, we designed a diet to mimick free-range AGS omega 6:3 ratios in captive AGS. Groups of wild-caught juvenile AGS were either fed: (1) Mazuri Rodent Chow (Standard Rodent chow, 4.95 omega 6:3 ratio), or (2) balanced omega 6:3 chow (Balanced Diet, 1.38 omega 6:3). AGS fed the Balanced Diet had plasma omega 6:3 ratios that mimicked plasma profiles of wild AGS. Balanced Diet increased female body mass before hibernation, but did not influence levels of cortisol in plasma or levels of the lipid peroxidation product 4-HNE in brown adipose tissue. Overall, as the mass gain is critical during pre-hibernation for obligate hibernators, the results show that mimicking a fatty acid profile of wild AGS facilitates sex-dependent mass accumulation without increasing stress indicators.

## Introduction

Hibernation is a regulated phenomenon where animals severely depress their metabolism and body temperature (Barnes 1989; Carey et al. 2003; Heldmaier et al. 2004; Jastroch et al. 2016). This evolutionary adaptation allows for survival without food for prolonged periods of time with no apparent ill effect (Carey et al. 2003; Drew et al. 2001). Arctic ground squirrels, *Urocitellus parryii* (AGS), are extensively studied due to their resilience to injury, duration of the hibernation season (up to 8 months for females), and extremely low torpid body temperature (Barnes 1989; Bogren et al. 2014, 2016).

Fatty acids, which are nutrients as well as fundamental structural elements of cellular biology, are carboxylic acids with hydrocarbon chains. Polyunsaturated fatty acids (PUFAs) have more than one double carbon bond, monounsaturated fatty acids (MUFAs) have a single double carbon bond, and saturated fatty acids (SFAs) have no double carbon bonds. PUFAs, such as the omega 3 PUFA alphalinolenic acid (ALA) and the omega 6 PUFA linoleic acid (LA) are not produced endogenously and must be obtained through diet. Multiple hibernating species of mammals selectively optimize PUFA consumption during the active season (Frank 1994; Munro and Thomas 2004). Omega 6 PUFAs are well documented for enhancing hibernation behavior in many species; specific levels of LA increase the depth of torpor and extend the time in torpor (Frank 2002; Frank et al. 2008; Frank and Storey 1995; Geiser and Kenagy 1993; Ruf and Arnold 2008). When diets lack essential PUFAs, time in torpor is significantly reduced resulting in loss of body mass (Florant et al. 1993), which affects the survival rate of free-range ground squirrels (Murie and Boag 1984).

Omega 3 PUFAs have been less studied in hibernation (Frank et al. 2004; Rice et al. 2021) with some results showing a potential negative influence on hibernation (Giroud et al. 2018; Hill and Florant 2000; Logan et al. 2020). Overall, two major perspectives exist for the relationship between omega 6 and omega 3 PUFAs and their influence on hibernation: (1) the ratio between omega 6 and omega 3 PUFAs may influence the molecular function of cardiac phospholipid membrane proteins such as the sacro-endoplasmic reticulum Ca^2+^-ATPase (SERCA) and therefore potentially impact hibernation behavior (Arnold et al. 2015; Giroud et al. 2013, 2018; Munro and Thomas 2004); and (2) there is no physiological difference between feeding omega 3 and omega 6 PUFAs on torpor (Frank et al. 2004, 2008). Our lab recently found higher levels of dietary omega 3 PUFAs with more balanced omega 6:3 ratios resulted in no ill effect on torpor bout length or time in hibernation in Arctic Ground Squirrels (AGS) but did increase brown adipose tissue (BAT) mass and torpid core body temperature (Rice et al. 2021).

While some captive studies point to negative influences from omega 3 PUFAs on hibernation, free-range studies show high levels of omega 3 PUFA consumption prior to hibernation in some species (Arnold et al. 2011; Florant et al. 1990), and dietary omega 3 PUFAs are known for their health benefits in humans, specifically in the reduction of pro-inflammatory cytokine production and cardiovascular risk (Lee et al. 2009; Zhao et al. 2007). Further, omega 6 and omega 3 PUFA products, such as eicosanoids, have opposing physiological functions (Schmitz and Ecker 2008). Omega 6 products, for example, series 2 prostaglandins, leukotrienes and cyclooxygenases, are often pro-inflammatory. Omega 3 products, such as series 3 leukotrienes, resolvins and neuroprotectin, are often anti-inflammatory (Schmitz and Ecker 2008). PUFAs additionally regulate inflammation by modifying intracellular signaling and gene expression. Feeding with a more balanced omega 6:3 diet has shown a reduction in cellular stress markers in non-hibernating species (Kearns et al. 1999; Lavie et al. 2009; Schmitz and Ecker 2008).

Common markers of physiological stress in some mammals (including AGS) include chronic increases in the production of cortisol (Sheriff et al. 2012) and 4-hydroxynonenal (4-HNE), a toxic end-product metabolically generated from omega 6 PUFA peroxidation (Awada et al. 2012; Schneiderman et al. 2005; Sheriff et al. 2012). Cortisol levels in ground squirrels follow the same seasonal patterns regardless of captivity or free-range location (Boonstra et al. 2001; Mateo 2006), making it a good marker to study in the laboratory. Increased dietary PUFA intake has been shown to influence cortisol release in humans (Gogus and Smith 2010; Mattson 2009; Yehuda et al. 2005). Chronic exposure to elevated cortisol causes suppression of the immune system, and may increase obesity, hyperglycemia, and hypertension, while high concentrations of 4-HNE are associated with cell toxicity and pathology of diseases such as atherosclerosis (Magomedova and Cummins 2016; Mattson 2009).

The first specific aim of this study was to measure the influence of feeding captive AGS a semi-synthetic diet that mimicks the omega 6:3 ratios found in free-ranging (termed wild) AGS on plasma fatty acid composition. The second specific aim of this study was to measure the impact of the omega 6:3 diet on cortisol, 4-HNE, and pre-hibernation weight gain in captive AGS. We first sampled free-range AGS plasma. We then formulated a diet that produces plasma omega 6:3 PUFA ratios in captive AGS (termed Balanced Diet) that most closely mimics the plasma omega 6:3 PUFA ratios of the free-ranging AGS. Given omega 3 PUFAs known health benefits, we predicted that mimicking a free-range diet (with Balanced Diet) will reduce markers of physiological stress and increase pre-hibernation mass gain in AGS.

## Materials and methods

### Trapping

The collection site for the wild live-trapped AGS was located on the northern side of the Brooks Range in Alaska (66° 38″ N, 149° 38″ W). In the summer and fall seasons prior to hibernation we set multiple traps baited with carrot slices to collect blood samples from free-ranging AGS that were released at a capture site after blood sampling. A subset of captured AGS was subsequently transported to the University of Alaska Fairbanks under Institutional Animal Care and use Committee (IACUC) approved protocols and Alaska Fish and Game permits.

### Animals and husbandry

All procedures were performed and approved in accordance with the University of Alaska Fairbanks IACUC and the Guide for Care and Use of Laboratory Animals (Eighth edition). The study was conducted during three years, each year separate groups of animals were used.

**Fig. 1:**
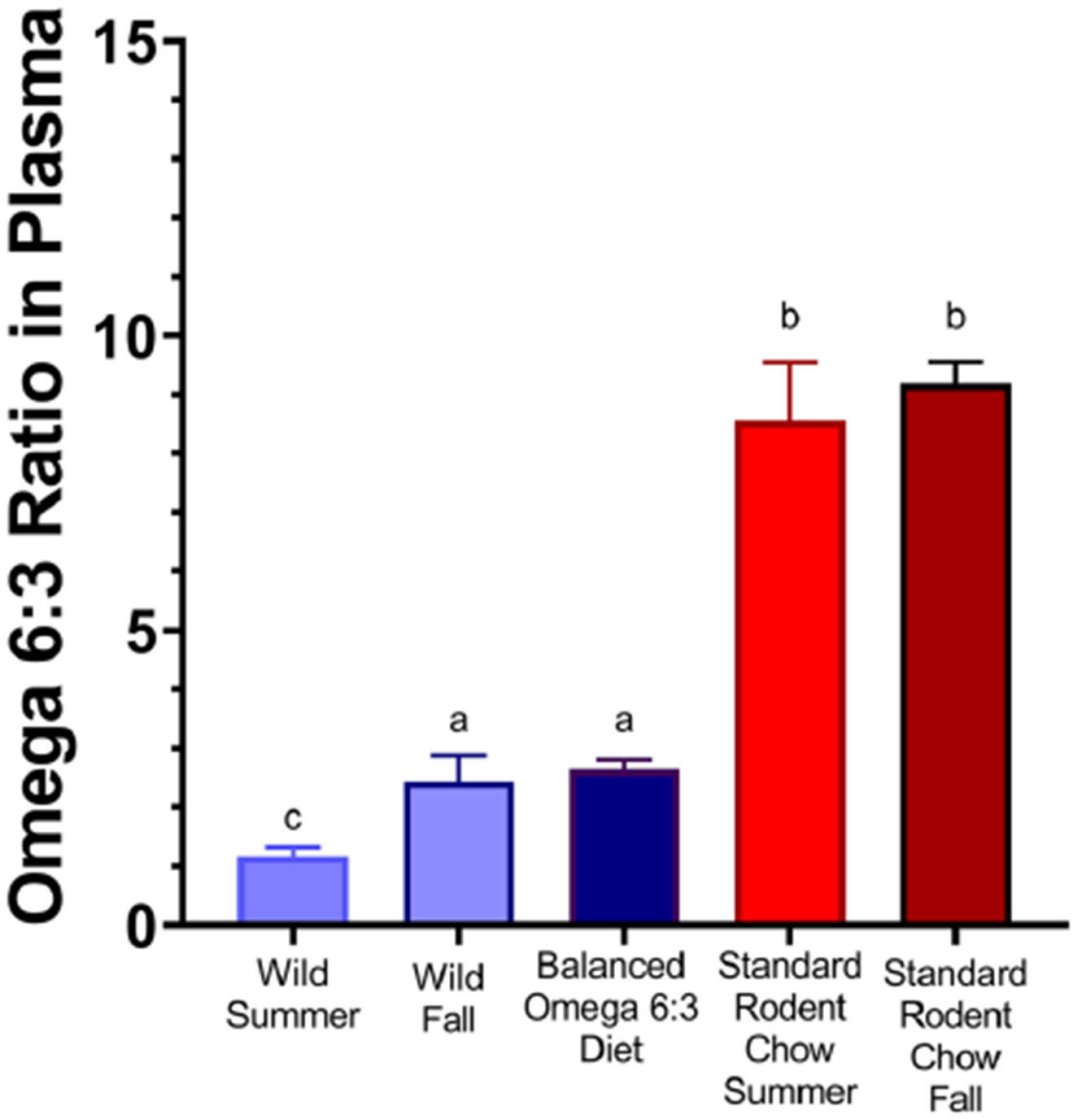
The ratios of omega 6:3 in plasma of wild AGS in summer (*n* = 9), wild AGS in early fall (*n* = 16) and fall captive Balanced Diet AGS (*n* = 9) are significantly lower than Standard Rodent Chow fed AGS in summer (*n* = 10) and early fall (AGS *n* = 18) (*p*<0.001, ANOVA, Tukey’s post-hoc test). Feeding a balanced omega 6:3 diet reduces the omega 6:3 ratio in plasma compared to captive AGS fed Standard Rodent Chow and mimics the plasma lipid profile of wild AGS. Different superscripts (a or b) signify that groups are different (*p* <0.001). Data incorporates previously published data on Fall Balanced Diet (*n* = 9) and Fall Standard Rodent Chow AGS (*n*=9) (Rice et al. 2021). Summer sampling occurred in very early July and fall sampling occurred in August, autumn time in the Arctic. Data shown are means ± SEM

In the first two years of the study, we examine the effects of diets on plasma fatty acid composition. Captive AGS were fed either Standard Rodent Chow (summer *n* = 10, fall *n* = 18) or Balanced Diet (fall *n* = 9) for the duration of two weeks prior to blood sampling. Wild AGS were sampled for blood in the corresponding season (summer *n* = 9, fall *n* = 16) (Fig. 1 and Table 2), utilizing AGS of mixed sexes and ages.

**Fig. 2:**
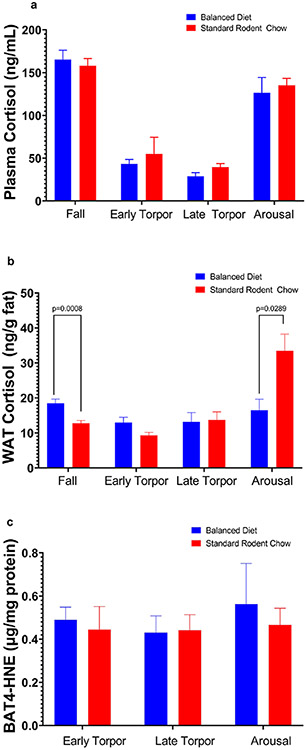
Diet did not influence cortisol in plasma or 4-HNE in brown adipose tissue (BAT), but influenced white adipose tissue (WAT) cortisol **a** Plasma cortisol measured in pre-hibernation and various stages of hibernation (early torpor, late torpor and arousal) did not differ between AGS fed either Standard Rodent Chow or Balanced Diet (mixed-effects analysis, FDR corrected). **b** Cortisol levels in WAT were higher in AGS fed the Balanced Diet prior to hibernation, but higher in Standard Rodent Chow AGS during the arousal phase of hibernation (*p<0.05, mixed-effects analysis, FDR corrected). **c** Neither diet significantly increased 4-hydroxynonenal (4-HNE), a marker of lipid peroxidation, in AGS BAT (two-way ANOVA, FDR corrected). Some AGS 4-HNE measurements fell slightly below the standard curve base. No significant differences were found between sexes, the data shown are means ± SEM. Fall Standard Rodent Chow *n* = 11, Fall Balanced Diet *n* = 12; Early Torpor, Late Torpor and Arousal are *n* = 3–4 per diet

**Fig. 3:**
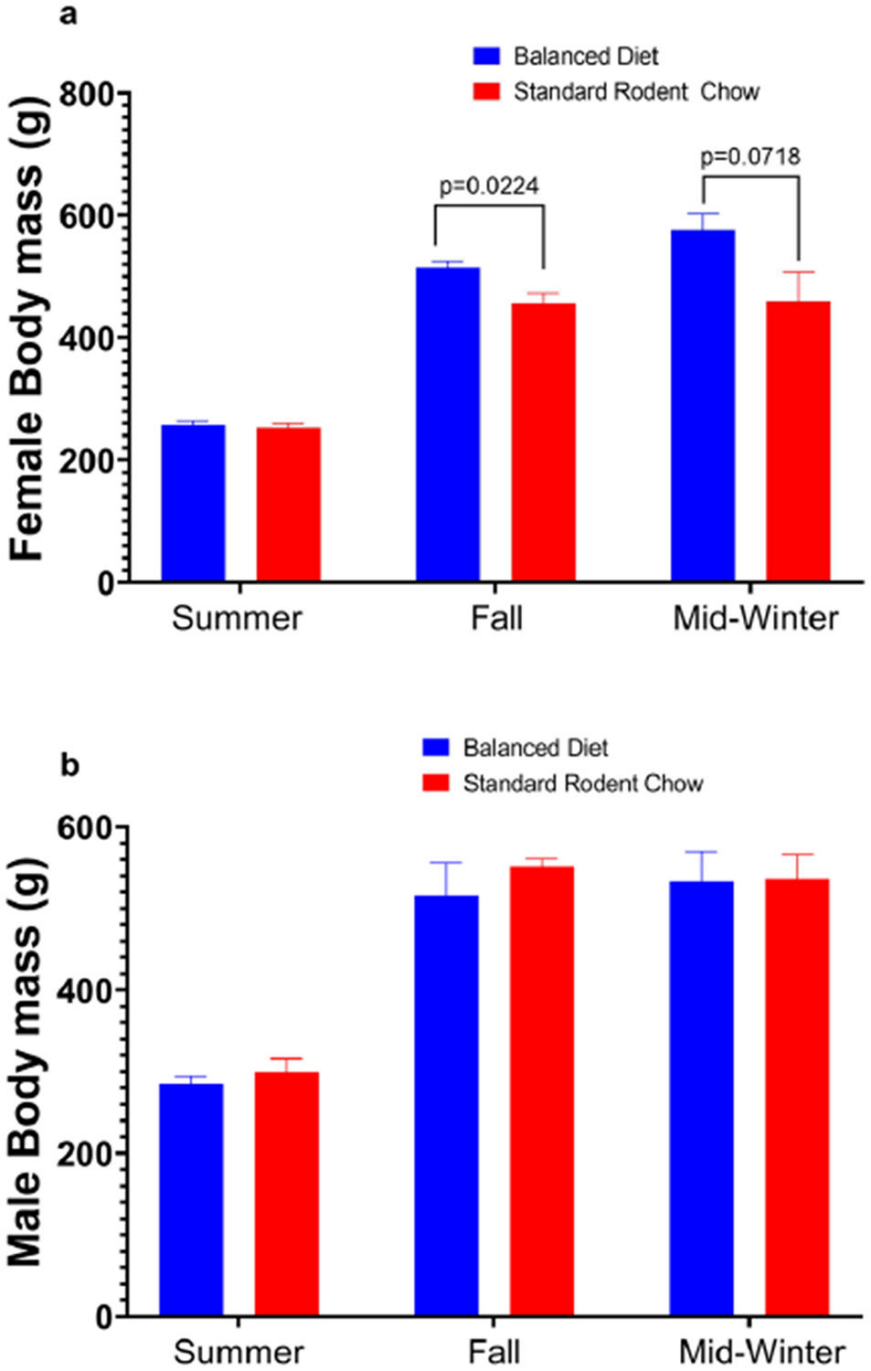
**a** The average body mass of females shows a significant increase in pre-hibernation for a Balanced Diet compared to Standard Rodent Chow AGS (*p<0.05, two-way repeated measures, FDR corrected). **b** The average body mass of males tracked before and during the hibernation season do not differ significantly between the two diets (two-way repeated measures, FDR corrected). Data shown are means ± SEM, Standard Rodent Chow *n* = 11, Balanced Diet *n* = 12

In the third year, we examined the effects of diets on cortisol and 4-HNE production and changes in body mass during the fall and hibernation periods (Figs. 2, 3). In this experiment, we used juvenile captive AGS fed either the Balanced Diet (*n* = 12, 6 females and 6 males) or Standard Rodent Chow (*n* = 11, 5 females and 6 males). Feeding trials started in mid-July, with tissue sampling (see below) in early-mid August and December. Animal body masses were measured in summer (prior to the start of feed trial), fall (~ three weeks into feed trial) and winter (~ three months into hibernation) with a digital scale accurate to 1 g (Escali).

Captive AGS were housed individually in 12″ × 19″ × 12″ stainless steel wire mesh with cotton bedding hanging cages over ammonia absorbing corn cob litter at ambient temperature (*T*_a_, 16–18 °C) and 16L:8D hour light/dark cycle until August 15, when they were moved to cold chambers with *T*_a_ of 2 °C at a 4L:20D hour light/dark cycle. Starting two weeks after capture and quarantine, mid-July animals were offered 47 g daily of either Standard Rodent Chow (#5663, Mazuri, PMI Nutrition International, Richmond, IN) or Balanced Diet (9GU5, formulated with Lab Diet, St. Louis, MO) and were provided water ad litbitum during the euthermic period as previously described (Rice et al. 2021). For animals kept through the hibernation season (year 3): once animals exhibited hibernation behavior, defined as the respiratory rate below 5 breaths per minute, inactivity and curled posture, food was withdrawn and animals were placed in polycarbonate cages (8.5″ × 17″ × 8.5″) with shavings, cotton bedding and gel hydration packets. The majority of AGS entered hibernation in September. Animal locations in the hibernation chamber were evenly staggered on racks between feed groups to ensure microenvironmental changes in the cold chamber would not proportionally impact one group over another. Care was taken to minimize all disturbances in the hibernation chamber during the hibernation season and access was restricted to research staff per University of Alaska Fairbanks IACUC approved protocols.

### Diet

Both diets were stored at −4 °C. The contents of the Balanced diet (9GU5, formulated by Lab Diet, St. Louis, MO) provided by the manufacturer were 23.4% protein, 6.5% fat, 6% ash, 3.3% fiber. A full list of ingredients is listed in Supplemental Table 2.

The contents of the Standard Rodent Chow (#5663, Mazuri, PMI Nutrition International, Richmond, IN) provided by the manufacturer were 23% protein, 6.5% fat, 4.5% fiber, 6.5% ash. A full list of ingredients is listed in Supplemental Table 2. We also provide a fatty acid composition of the diets determined by GC in the result section (Table 1d) as previously described (Rice et al. 2021).

### Summer and fall blood collection

Blood was sampled from captive and free-ranging euthermic AGS via toenail clip in early July (summer) and early-mid August (autumn in the Arctic) (Table 1a, b). After blood was sampled, the toenail was cauterized utilizing a caustic pencil (Silver Nitrate). Once verified that blood had clotted, wild animals were released. Blood was immediately centrifuged and plasma samples were immediately put on dry ice until transfer to −80 °C storage. Wild and captive fall sampling of AGS occurred over two years, measurements were combined because FDR corrected t-tests between groups showed no significant differences between years.

### Continuous monitoring of hibernation state

Samples analyzing stress indicators (4-HNE and cortisol) came from AGS that were implanted with I-button temperature loggers (Maxim Integrated, San Jose, CA) using sterile technique in the abdominal cavity in early August from another study as previously described (Rice et al. 2021). Animals were anesthetized with isoflurane (5% mixed with medical grade oxygen and delivered at 1.5 L/min) to achieve a surgical plane of anesthesia and maintained on 2.5–3% isoflurane throughout the procedure as previously described (Rice et al. 2021). During the hibernation season, the “shavings added” method was employed to track torpor bout length. On the first day of torpor when respirations were below 5 breaths per minute and the animal was inactive and showed a curled posture, shavings were placed onto the animal’s back. Using this method to identify when animals were hibernating, we monitored the animals daily through the hibernation season. End of a torpor bout was determined when animals’ shavings were disturbed, signifying an interbout arousal (IBA).

### Tissue collection

Fall visceral white adipose tissue (WAT) samples were biopsied during the implantation of I-button transmitters. The animals began their designated diets approximately three weeks prior to fall WAT sampling (Table 1c). Tissues were collected in mid-December at three distinct physiological states of the torpor bout: early torpor (defined as 10–25% of torpor bout completed based on the average of the previous two torpor bouts, *n* = 7), late torpor (defined as 89–100% of torpor bout completed based on the average of the previous two torpor bouts, *n* = 8) and arousal (core body temperature above 33 °C, *n* = 8) as previously described (Rice et al. 2021). Arousal was induced from torpor by handling at 9 am and arousal tissues were collected at 1 pm in December. Arousal in AGS typically takes four hours and all arousal animals were 33 °C or above when euthanized. All hibernating animals were euthanized between 9–11 am in December. Prior to tissue collection, euthermic animals were anesthetized with isoflurane (5% mixed with medical grade oxygen and delivered at 1.5 L/min) to achieve a surgical plane of anesthesia. Blood was sampled by cardiac puncture within 3 min after removing AGS from the home cage. Animals were immediately decapitated after blood sampling and tissues were dissected, wrapped in foil and placed immediately on dry ice.

### Lipid fatty acid analysis

100 μl of plasma was thawed and decanted into a screw-top test tube. 100 μg of 19:0 free fatty acid was added as an internal fatty acid standard (odd chain fatty acids are found in low abundance in non-rumen animals). Plasma lipids were then converted to fatty acid methyl esters with the addition of sulfuric acid in methanol and heated for 60 min at 100 °C. Fatty acid methyl esters were extracted from sample tubes with the addition of water and distilled petroleum ether (mixed hexanes). The resulting non-polar ether phase was decanted and dried down under a gentle stream of nitrogen at ambient temperature.

Dried samples were immediately reconstituted with 100 μL of petroleum ether and decanted to a sample vial. Fatty acid methyl esters were then analyzed with a Shimadzu gas chromatograph (GC) model 2010. Samples were injected in split mode into a Restek (Bellefonte, PA) FAMEWAX 30 m column. The GC was programmed from 160 to 220 °C and detection was conducted with flame ionization detection (FID). Chromatograms and data were reviewed and calculated with Shimadzu Class VP software. Data are expressed in percent of fatty acid per ml of plasma. Calculation of total omega 9 fatty acids predominately comes from oleic acid (18:1ω9), but also includes very small contributions from 20:1ω9, 20:3ω9 and 22:1ω9 (all under 1% of total fatty acid percentage). Calculation of total omega 6 PUFAs predominately comes from linoleic acid (18:2ω6) and arachidonic acid (20:4ω6), but also includes very small contributions from 18:3ω6, 20:2ω6, 20:3ω6, and 22:4ω6 and 22:5ω6 (all under 1% total fatty acid percentage). Calculation of total omega 3 PUFAs predominately comes from alpha-linolenic acid (18:3ω3), DHA (22:6ω3), DPA (22:5ω3) and EPA (20:5ω3), but also includes very small contributions from 18:4ω3, 20.3ω3 and 20:4ω3 (all under 1% total fatty acid percentage).

### Cortisol analysis

Plasma (50 μL per sample) and white adipose tissue (WAT, 25 mg, wet weight per sample) were analyzed following a solid phase extraction (SPE, using Bond Elut 6 mL C18 columns) protocol (Newman et al. 2008). Briefly, homogenized WAT samples were extracted in methanol (HPLC-grade, Fisher Scientific, Waltham, MA, USA) by 15 min sonication and subsequent 1 h shaking in a Multi-Pulse Vortexer. Extracts were centrifuged at 2000 rpm for 10 min at 4 °C and the supernatant collected for SPE elution following the protocol (Newman et al. 2008). Plasma samples were diluted with 10 mL of dH_2_O and submitted for SPE elution. The SPE eluates were dried in a water bath at 40 °C under an air stream, reconstituted in phosphate-buffered-saline with gelatin (PBSG) buffer and analyzed in a radioimmunoassay (Wingfield and Farner 1975) using a Sigma-Aldrich antibody (C8409, Saint Louis, MO, USA), intra-assay and inter-assay CV < 2%. To control for loss of cortisol during the extraction process 2000 cpm of tritiated cortisol (cortisol labeled with radioactive hydrogen, PerkinElmer, Boston, MA, USA) was added to each sample and final cortisol titers were adjusted for the sample-specific % recovery (mean = 71% for WAT, and 84% for plasma). After SPE, serially diluted AGS WAT and plasma samples yielded displacement curves that are parallel to the cortisol standard curve. Assay results were normalized by converting to units of ng/g (WAT) and ng/mL (plasma). Results of a *t*-test did not indicate a statistically significant effect of sex on cortisol concentrations and therefore sex of individuals was not included in further statistical analyses.

### Lipid peroxidation analysis

10 mg of brown adipose tissue (BAT) samples were homogenized with a dounce homogenizer and 200 μL RIPA buffer. Samples were centrifuged at 4 °C and pelleted down. Supernatants were centrifuged again at 4 °C, lysates were frozen at −80 °C. Lysates were diluted 2× and run with a 4-hydroxynonenal (4-HNE) assay kit (ab238538, Abcam, Cambridge, MA) on an Epoch microplate reader (BioTek Inc., Winooski, VT) at an absorbance of 450 nm in duplicates and results were plotted along a standard curve. Lysate protein concentration was calculated with a Pierce BCA Protein kit (ThermoScientific, Rockford, Il). Lysates were diluted by 4× and 2% SDS was added to samples to limit interference by lipids (Kessler and Fanestil 1986). Samples were measured on an Epoch microplate reader (BioTek Inc., Winooski, VT) at an absorbance of 562 nm in duplicates and results were plotted along a standard curve. We selected BAT to measure 4-HNE due to its high metabolic activity in hibernation. Recent data may indicate metabolically active tissues such as BAT might have higher levels of molecular damage over hibernation in AGS (Wilbur et al. 2019) and our lab has shown BAT is the only tissue to show oxidative stress after arousal (Orr et al. 2009).

### Statistical analysis

Results were analyzed using SPSS statistical package (v.25) and GraphPad Prism 9 (v9.3.1). Data are shown as mean ± standard error of the means (SEM). Data were checked for normal distribution using the chi-squared goodness-of-fit test. Effects of diet and captive conditions on the fatty acid profile were assessed using one-way ANOVA and Tukey HSD post-hoc analysis. Cortisol statistical comparisons were conducted using a mixed-effects model (with the individual as a random effect) with FDR corrections. 4-HNE measurements were assessed with two-way ANOVA with FDR corrections and body masses were assessed using two-way repeated measured with FDR corrections. For ANOVA tests that violate the assumption of homogeneity of variances, a Welch ANOVA was used. Graphs were produced with GraphPad Prism 9 (v9.3.1).

## Results

### Differences in omega 6:3 PUFA profiles between captive and wild arctic ground squirrels can be ameliorated with diet

Plasma fatty acid (FA) profiles were compared between wild AGS and captive AGS (Fig. 1). The ratio of omega 6:3 PUFAs was significantly higher in captive AGS fed Standard Rodent Chow compared to wild (free-ranging) AGS (*p* < 0.001, ANOVA, Fig. 1). Subsequently, we formulated a diet based on the wild omega 6:3 ratio termed Balanced Diet. Balanced Diet contains a 1.38:1 ratio of omega 6 to omega 3 PUFAs, compared to the 4.95:1 ratio of Standard Rodent chow (Table 1d). Both diets contained the same amount of overall fat and both diets fell within the recommended overall PUFA range determined from previous studies for hibernating squirrels (33–74 mg/g) (Frank et al. 2008). The main source of omega 3 PUFA in the Balanced Diet was alpha-linolenic acid (ALA), which came from the addition of flaxseed oil (Table 1d, Supplemental Table 2). When captive AGS were fed Balanced Diet, the plasma omega 6:3 ratio maintains the omega 6:3 ratio seen in wild AGS sampled during the fall season (Fig. 1).

Overall, the analysis showed higher percentages of omega 3 PUFAs in the plasma of wild and AGS fed Balanced Diet than in Standard Rodent Chow fed AGS in fall (*p* < 0.05, ANOVA, Table 2). ALA is the parent compound of the omega 3 PUFA cascade products eicosapentaenoic acid (EPA), docosapentaenoic acid (DPA) and docosahexaenoic acid (DHA). Balanced Diet ALA plasma levels were closer to wild AGS than Standard Rodent Chow fed AGS, but still were significantly lower than wild AGS (*p* < 0.001, ANOVA, Table 2). Balanced Diet and Wild fed AGS DHA and EPA were significantly greater than Standard Rodent Chow fed AGS, while Balanced Diet fed AGS DHA was significantly higher than wild AGS (*p* < 0.001, ANOVA, Table 2). DPA (ω3) concentrations of wild AGS were approximately 5 times higher than in AGS fed either captive diet (Balanced Diet or Standard Rodent Chow) (*p* < 0.001, ANOVA, Table 2).

Plasma omega 3 PUFA decreased slightly in the wild from summer to fall (*p* < 0.05, FDR corrected, *t*-test, Supp. Table 1). There was a corresponding slight, non-significant increase in fall omega 6 PUFA in wild AGS plasma (*p* = 0.129). In captive Standard Rodent Chow fed AGS, no fatty acid levels statistically changed from summer to fall (Supp. Table 1). Overall, the seasonal increase in plasma omega 6:3 ratios from summer (1.2:1) to fall (2.4:1) in wild AGS (Fig. 1) appears due to decreased omega 3 PUFA and a slight increase in omega 6 PUFA (Supp. Table 1). The Balanced Diet fed AGS best reflects this fall wild omega 6:3 plasma ratio (Fig. 1).

While the percent of omega 6 PUFA did not differ significantly between Wild and Standard Rodent Chow in the fall, Balanced Diet animals had significantly less plasma omega 6 PUFA than both groups (*p* < 0.001, ANOVA, Table 2). Interestingly, arachidonic acid (ARA), a major omega 6 product, did not vary between any group while linoleic acid (LA) significantly differed between treatment groups (ANOVA, Table 2).

Other FAs beyond omega 3 and 6 PUFA were impacted by captivity. Wild AGS had a lower percent of omega 9 s as compared to captive AGS fed Standard Rodent Chow or Balanced Diet fed AGS (*p* < 0.001, ANOVA, Table 2). Oleic acid, the major omega 9 monounsaturated fatty acid (MUFA), followed this trend (Table 2).

### Balanced diet does not increase physiological stress marker or lipid peroxidation, but increases female body mass

Plasma cortisol, a marker of physiological stress, and brown adipose tissue (BAT) 4-HNE, a marker of oxidative stress and lipid peroxidation, did not differ between Balanced Diet and Standard Rodent Chow fed AGS, but plasma cortisol significantly changes between season and physiological state (*p* < 0.0005, mixed-effects model, Fig. 2a, c). White adipose tissue (WAT) cortisol was significantly higher in Balanced Diet fed animals in the fall prior to hibernation (approximately three weeks into the feed study) (*p* < 0.05, mixed-effects model, Fig. 2b). During arousal from torpor, however, WAT cortisol levels were significantly higher in Standard Rodent Chow fed AGS compared to Balanced Diet fed AGS (*p* < 0.05 mixed-effects model, Fig. 2b). WAT cortisol also significantly changes between season and physiological state (*p* < 0.0001, mixed-effects model, Fig. 2b). Body mass in males was not significantly different between diets, but female body mass did increase prior to hibernation in Balanced Diet animals compared to controls and trended toward an increase in hibernation (*p* < 0.05, two-way repeated measures, Fig. 3).

## Discussion

Here we show that free-range AGS have more balanced levels of omega 6 to omega 3 PUFAs compared to captive AGS fed Standard Rodent Chow, who have elevated levels of omega 6 PUFA and depressed levels of omega 3 PUFA. The ratio of omega 6:3 PUFA are not just slightly different between the diets; summer Standard Rodent Chow plasma is 7.3 times the ratio of wild AGS in summer. This suggests that the current laboratory diet, which is indicative of a Westernized diet with heavy dietary input from omega 6 PUFA and deficiencies in omega 3 PUFA (Simopoulos 2002), does not represent the wild ratio of omega 6:3. Feeding the Balanced Diet led to omega 6:3 ratios that mimicked plasma profiles of wild AGS in autumn. Mimicking the wild AGS fatty acid profile in captive AGS, with an increase in dietary omega 3 s and a greater balance between omega 6:3 PUFAs, did not lead to increased physiological stress markers, such as plasma glucocorticoid production, and peroxidative damage in captive AGS, but did increase female mass gain. Further, we previously have shown feeding a balanced 6:3 diet in captive AGS did not reduce time in torpor, torpor bout length or delay seasonal entrance to hibernation, but rather increased brown adipose tissue (BAT) and torpid core body temperature (Rice et al. 2021).

An advantage to mimicking wild omega 6:3 ratios in captive hibernators may be the sex-dependent mass gain in Balanced Diet animals. We found significantly higher body mass prior to hibernation in female Balanced Diet animals compared to Standard Rodent Chow animals. Obligate hibernators, such as AGS, prepare for hibernation by increasing body mass (Galster and Morrison 1976; Sheriff et al. 2013). Mass increase is predominately fat, with both females and males significantly increasing fat mass in August and September, but not lean mass (Sheriff et al. 2013). Mass gain prior to hibernation is correlated with entrance into hibernation as well as survival rates (Humphries et al. 2003; Morton et al. 1974; Murie and Boag 1984). While the timing of entrance into hibernation was not significantly impacted by diet as our lab previously showed (Rice et al. 2021), the Balanced Diet-induced increase in female mass gain prior to hibernation, and evidence that mass gain is critical to hibernation physiology (Galster and Morrison 1976; Sheriff et al. 2013), could indicate a novel, beneficial effect of a diet mimicking wild fatty acid ratios in captive animals. This is especially important in regards to juvenile ground squirrels, which are known to triple their mass over the active season (Boag and Murie 1981). In non-hibernators, studies have found fat mass sexual dimorphisms in response to a high-fat diet (Amengual-Cladera et al. 2012). Curiously, sex-dependent impacts from omega 3 PUFAs in humans lead to weight loss, not weight gain (Munro and Garg 2013). The potential mechanism of action for a Balanced Diet increasing mass gain in females is an intriguing question.

Pre-hibernation mass increase in Balanced Diet could be linked to WAT cortisol levels. Cortisol production occurs in both the adrenals and white adipose tissue (via enzymatically converting cortisone) (Andrew et al. 2005; Magomesdova and Cummins 2016). Evidence suggests cortisol is the primary glucocorticoid in AGS (Boonstra et al. 2001). While often used as an index for physiologic stress, cortisol plays a regulatory role in multiple physiological processes and is a major regulator of carbohydrate, fat and protein metabolism (Magomedova and Cummins 2016). Importantly, glucocorticoids can enhance and regulate adipogenesis and fat deposition, potentially representing a mechanism for mass gain in pre-hibernation Balanced Diet animals (Lee et al. 2014; Sheriff et al. 2012). However, a significant (albeit moderate ~ 40%) increase in WAT cortisol in animals on a Balanced diet during pre-hibernation was not a result of higher circulating (in blood plasma) levels of that hormone suggesting a conversion of cortisone to the active glucocorticoid cortisol via the 11-hydroxysteroid dehydrogenase type 1 pathway in fat tissues (Andrew et al. 2005; Bujalska et al. 1997). Whether such a moderate increase of cortisol in white adipose tissues was associated with an increased blood flow in WAT and enhanced adipogenesis (as evident in higher body mass of females) in response to Balanced Diet is currently unknown and calls for further investigation. In support of this possibility, WAT cortisol levels in torpid animals (with a reduced blood flow in white adipose tissues) were not different between the dietary groups.

Currently, a contribution of cortisol produced in white adipose tissues to a systemic increase of this hormone in the general blood circulation, and subsequent detrimental effects of such elevated cortisol exposure, is a subject of debate (Aldhahi et al. 2004; Andrew et al. 2005; Basu et al. 2006). However, all previous studies have been conducted on non-hibernating species and little is known about the magnitude and metabolic effects of cortisol release from the splanchnic region in hibernators (which contains the major abdominal WAT deposits). Curiously, we observed a dramatic two-fold increase of WAT cortisol levels in Standard Rodent Chow fed AGS, but not in Balanced Diet fed AGS, during the arousal phase of hibernation. Plasma cortisol levels also increased in arousal, but did not significantly differ between dietary treatments. An interpretation of these differing patterns of cortisol concentrations in blood plasma versus WAT tissues is limited by small sample sizes. However, it seems unlikely that an increase in plasma cortisol levels was due to production in splanchnic tissues and likely reflects increased synthesis of hormone in the adrenals during the arousal phase of hibernation. There is, however, a possibility that feeding animals with a Standard Rodent Chow diet might have detrimental delayed effects during post-hibernation, when ground squirrel reliance on endogenous reserves is critical (Barnes 1984; Sheriff et al. 2011). Smaller endogenous fat stores may be depleted faster than in animals fed Balanced diet (this study), and chronically increased cortisol levels are likely to increase gluconeogenesis leading to loss of muscle proteins and suppression of the immune system (Gelfand et al. 1984; Khani and Tayek 2001). Further research on the effects of a Balanced Diet during the post-hibernation period is warranted, as potential costs and benefits of a Balanced Diet might be more apparent during post-hibernation.

We originally hypothesized Balanced Diet would reduce markers of physiologic stress and lipid peroxidation in captive AGS. Previous research shows BAT lipid peroxidation between two species fed the same PUFA diets did not differ (Harlow and Frank 2001), but when ground squirrels were fed high levels of omega 6 PUFA BAT lipid peroxidation did increase (Frank and Storey 1995). Plasma cortisol levels and BAT 4-HNE, a lipid peroxidation product, did not differ between diets in our study. The fact that free-range AGS plasma presents high levels of ALA compared to most laboratory diets and our previous findings that feeding a Balanced Diet did not reduce time in torpor, delay seasonal entrance to hibernation or reduce torpor bout length, may indicate that omega 3 PUFAs, and diets that mimic wild omega 6:3 ratios, are not inherently harmful to Arctic Ground Squirrels.

There is, however, variability in the outcomes of omega 3 PUFA studies on hibernators. Multiple variables distinguish these studies, such as the employment of differing sources of omega 3 PUFAs (linseed oil, menhaden oil, flaxseed oil), differing dietary ratios of omega 6:3 and differing species (Frank et al. 2004; Giroud et al. 2018; Hill and Florant 2000; Logan et al. 2020; Rice et al. 2021). To note, results of an ill-effect from feeding hibernators omega 3 PUFAs, specifically an inhibition of hibernation (Hill and Florant 2000) and an increase in some inflammatory markers (Logan et al. 2020), came from diets that were not balanced between omega 6 and omega 3 PUFAs; rather, dietary omega 3 PUFAs were higher than the omega 6 PUFAs. While one hypothesis could be differences are species specific, we hypothesize previous findings of a negative influence from omega 3 PUFAs may stem from an oversupply of dietary omega 3 PUFAs or an undersupply of omega 6 PUFAs, which Logan et al. supports (Logan et al. 2020). An outlier to this observation would be the delay in hibernation onset in dormice from dietary omega 6:3 ratios of 2.92:1, which also noted no change in body mass, temperature and hibernation patterns between feed groups (Giroud et al. 2018). Giroud et. al did supplement with a fish oil heavy in DHA whereas we used flaxseed oil, heavy in ALA. Giroud and co-authors hypothesized a fast removal of DHA from phospholipid membranes prior to entering hibernation may have played a role in delayed hibernation in the dormice. Last, but not least, subtle differences still exist between our Balanced Diet and wild AGS plasma PUFA profiles, such as differences in DPA levels or the fact wild AGS had a lower percent of omega 9s compared to captive AGS. Future studies would be needed to elucidate the more subtle nuances of specific diets and any physiological role they may play in hibernation.

One could point to the higher torpid core body temperature (T_b_) from feeding omega 3 PUFAs that our lab previously documented (Rice et al. 2021) as a potential negative toward AGS hibernation. While elevated T_b_ has been considered in an adverse light in that it is typically associated with shorter torpor bouts and higher consumption of energy, there may be an argument that elevated T_b_ could allow for increased basal rates of multiple protective and tissue repair mechanisms in torpor. Specific molecular processes like protein synthesis are dependent on euthermic temperatures above 18 °C (van Breukelen and Martin 2001), but other cellular repair mechanisms could hypothetically increase as a function of temperature. Recent studies have supported this possibility with hibernators kept at warmer temperatures displaying less telomere damage (Nowack et al. 2019). Importantly, the relevance of the small *T*_b_ difference reported by Rice et al. 2021 may be minor since 80–90% of the energy (depot lipid) utilized during hibernation is during arousal episodes (Karpovich et al. 2009), and these two diet groups did not significantly differ in arousal episode frequency. One caveat of our study is the impact of diet on seasonal exit from hibernation could not be measured. Theoretically, animals with higher *T*_b_ may deplete seasonal fuel resources faster than controls. However, the increased mass of females on a Balanced Diet could represent greater fuel resources at the on-set of hibernation than the control animals just for this reason.

In conclusion, our results show profoundly different omega 6:3 PUFA ratios in wild animals compared to captive AGS fed Standard Rodent Chow. Our results show that we can ameliorate these differences with a Balanced Diet and that no apparent physiological stress resulted from feeding this diet. Additionally, the increase in female mass gain prior to hibernation from Balanced Diet may represent a physiological advantage to mimicking wild omega 6:3 ratios in captive animals. We conclude omega 3s are not inherently harmful to hibernation in AGS and that ratios of omega 6:3 PUFAs may be considered in the formulation of future hibernator feed in captivity, which would provide a better imitation of the nutritional state of wild animals to investigate their physiology during hibernation under captive conditions.

## Supplementary Material

Supplementary Material

## Figures and Tables

**Table 1:** An overview of workflow

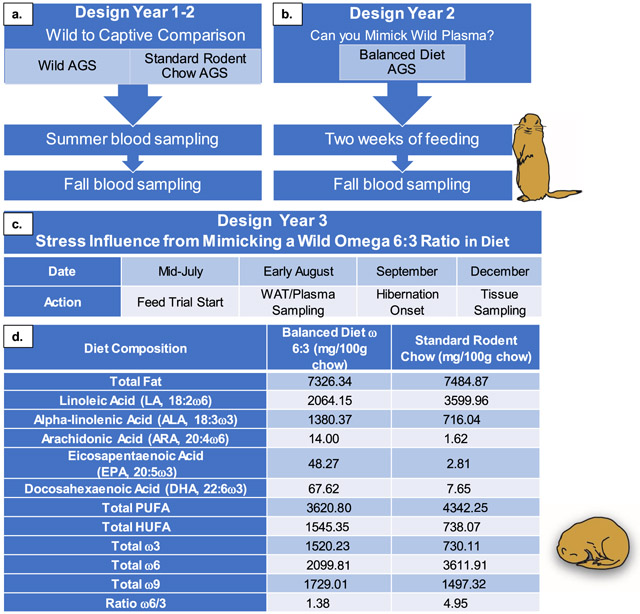

a. Year 1 compared captive AGS fed Standard Rodent Chow (#5663, Mazuri) plasma fatty acids to wild AGS in summer and fall b. Year 2 tested if Balanced Diet (9GU5, Lab Diet) could produce plasma fatty acids levels that mimicked a wild plasma profile c. Year 3 tested the stress impacts of mimicking a wild omega 6:3 diet in captive AGS. d. Concentration of fatty acids in Balanced ω 6:3 Chow (9GU5, Lab Diet) and Standard Rodent Chow (#5663, Mazuri) diets. The values listed are in mg/100 g chow. Balanced Diet was formulated based on wild AGS fatty acid profiles during fall (Fig. 1). HUFA stands for “highly unsaturated fatty acids” and PUFA stands for “polyunsaturated fatty acids”

**Table 2:** Plasma Omega 3 PUFAs are significantly higher in fall wild AGS and balanced diet fed AGS than standard rodent chow fed AGS

Fatty acid (percent total fatty acid)	Plasma: wild AGS in fall	Plasma: balanced Omega 6:3 diet fed AGS in fall	Plasma: standard rodent chow fed AGS in fall
Eicosapentaenoic acid (EPA, 20:5ω3)	2.50 ± 0.29^a^	2.05 ± 0.12^a^	0.38 ± 0.04^b^
Docosapentaenoic acid (DPA, 22:5ω3)	2.47 ± 0.18^a^	0.63 ± 0.58^b^	0.49 ± 0.03^b^
Docosahexaenoic acid (DHA, 22:6ω3)	1.23 ± 0.08^a^	2.04 ± 0.14^b^	0.56 ± 0.07^c^
Alpha linolenic acid (ALA, 18:3ω3)	11.30 ± 1.25^a^	5.15 ± 0.37^b^	2.36 ± 0.09^b^
Arachidonic acid (ARA, 20:4ω6)	6.21 ± 0.72^a^	4.22 ± 0.47^a^	4.86 ± 0.43^a^
Linoleic acid (LA, 18:2ω6)	27.23 ± 1.18^a^	21.17 ± 0.72^b^	30.06 ± 1.11^a^
Oleic acid (18:1ω9)	12.59 ± 1.62^a^	26.68 ± 1.17^b^	26.46 ± 1.92^b^
Total Omega 3	18.44 ± 1.62^a^	10.23 ± 0.30^b^	3.99 ± 0.10^c^
Total Omega 6	35.52 ± 1.86^a^	26.85 ± 1.21^b^	36.57 ± 1.56^a^
Total Omega 9	13.08 ± 1.64^a^	27.17 ± 1.18^b^	26.94 ± 1.93^b^

Data shown are the percent of total plasma fatty acid (FA) in wild AGS and captive AGS fed Standard Rodent Chow or a Balanced omega 6:3 Diet (Fall Wild (*n* = 16), Fall Standard Rodent Chow (*n* = 18) and Fall Balanced Omega 6:3 Diet Captive AGS (*n* = 9)). Omega 6:3 ratios are shown in Fig. 1. Letters (a or b) signify *p* < 0.05 between feed groups (if letters are the same there is no difference between groups) (ANOVA, Tukey HSD post-hoc test). Data incorporates previously published data on Fall Balanced Diet (*n* = 9) and Fall Standard Rodent Chow AGS (*n* = 9) (Rice et al. 2021). Data shown are means ± SEM
