# Supplementary material for: Translating PUFA omega 6:3 ratios from wild to captive hibernators (Urocitellus parryii) enhances sex-dependent mass-gain without increasing physiological stress indicators
Source: J Comp Physiol B. Author manuscript; Available in PMC 2022 Jul 1. (PMC9197884; doi:10.1007/s00360-022-01437-6)
Supplement: Supplementary Material [file NIHMS1806807-supplement-Supplementary_Material.pdf]

<u>Supplemental Table for</u>: Translating PUFA Omega 6:3 ratios from Free Range to Captive Hibernators (*Urocitellus parryii*) Enhances Sex-Dependent Weight-Gain Without Stress Indicators

Authors: Monica Mikes[2#], Sarah A. Rice[1,2*#], Doug Bibus[3], Alexander Kitaysky[2], Kelly L. Drew[1,2]
[1]Department of Chemistry and Biochemistry, University of Alaska Fairbanks, [2]Institute of Arctic Biology, University of Alaska Fairbanks, [3]Lipid Technologies, LLC

For Submission to: Comparative Physiology B

Supplemental Table 1

FATTY ACID	*Wild Summer*	*Wild Fall*	*Standard Rodent Chow Summer*	*Standard Rodent Chow Fall*
Eicosapentaenoic Acid (EPA, 20:5ω3)	4.36±0.05	2.50±0.29*	0.35±0.44	0.38±0.04
Docosapentaenoic Acid (DPA, 22:5ω3)	4.40±0.53	2.47±0.18*	0.59±0.10	0.49±0.03
Docosahexaenoic Acid (DHA, 22:6ω3)	2.22±0.11	1.23±0.08*	0.65±0.28	0.56±0.07
Alpha Linolenic Acid (ALA, 18:3ω3)	14.19±0.87	11.30±1.25	2.75±0.19	2.36±0.09
Arachidonic Acid (ARA, 20:4ω6)	4.63±0.83	6.21±0.72	5.14±0.49	4.86±0.43
Linoleic Acid (LA, 18:2ω6)	24.08±2.65	27.23±1.18	30.62±1.89	30.06±1.11
Oleic Acid (18:1ω9)	9.87±3.26	12.59±1.62	25.65±2.96	26.46±1.92
Total Omega 3	26.73±1.19	18.44±1.62*	4.51±0.25	3.99±0.10
Total Omega 6	30.26±2.99	35.52±1.86	37.43±2.83	36.57±1.56
Total Omega 9	10.23±3.28	13.08±1.64	26.12±2.99	26.94±1.93

Supplemental Table 1 Plasma Omega 3 fatty acids decrease seasonally in wild AGS. Data shown is percent of total plasma fatty acid (FA) in wild AGS and captive AGS fed Standard Rodent Chow (Summer Wild AGS (n=9), Summer Standard Rodent Chow (n=10), Fall Wild (n=16), Fall Standard Rodent Chow (n=18)). Asterisks signify difference between seasons in same feed groups ($p<0.05$, FDR corrected, t-test). Data shown are means ±SEM

Supplemental Table 2

Diet	Ingredients
Balanced Diet (9GU5)	Ground Corn, Dehulled Soybean Meal, Whole Wheat, Fish Meal, Wheat Middlings, Cane Molasses, Flaxseed Oil, Brewers Dried Yeast, Wheat Germ, Porcine Meat and Bone Meal, Ground Oats, Porcine Animal Fat Preserved With BHA and Citric Acid, Dried Beet Pulp, Dehydrated Alfalfa Meal, Dried Whey, Calcium Carbonate, Salt, Menadione Dimethylpyrimidinol Bisulfite (source of Vitamin K), Choline Chloride, Cholecalciferol, DL-Methionine, Vitamin A Acetate, Manganous Oxide, Zinc Oxide, Ferrous Carbonate, Copper Sulfate, Zinc Sulfate, Calcium Iodate, Calcium Carbonate, Cobalt Carbonate, Pyridoxine Hydrochloride, DL-Alpha Tocopheryl Acetate (Form of Vitamin E), Folic Acid, Thiamine Mononitrate, Nicotinic Acid, Calcium Pantothenate, Riboflavin Supplement, Vitamin B-12 Supplement
Standard Rodent Chow (Mazuri)	Dehulled soybean meal, Ground Corn, Wheat Middlings, Ground Wheat, Soybean Oil, Cane Molasses, Ground Oats, Calcium Carbonate, Dried Beet Pulp, Dehydrated Alfalfa Meal, Wheat Germ, Brewers Dried Yeast, Salt, Calcium Propionate, di-Calcium Phosphate, Pyridoxine Hydrochloride, DL-methionine, Choline Chloride, Dried Yucca Shidigera Extract, Menadione Sodium Bisulfite Complex (source of vitamin K), Vitamin D3 Supplement, Vitamin A Acetate, Folic Acid, Thiamine Mononitrate, D-alpha Tocopheryl Acetate (form of Vitamin E), Vitamin B12 Supplement, Niacin Supplement, Calcium Pantothenate, Riboflavin, Manganous Oxide, Zinc Oxide, Ferrous Carbonate, Copper Sulfate, Zinc Sulfate, Calcium Iodate, Cobalt Carbonate

Supplemental Table 2 Contents of Balanced Diet (9GU5, Test Diet) and Standard Rodent Chow (5663, Mazuri) provided by the manufacturers
